# Potential mechanisms of resistance to venetoclax and strategies to circumvent it

**DOI:** 10.1186/s12885-017-3383-5

**Published:** 2017-06-02

**Authors:** Stephen K. Tahir, Morey L. Smith, Paul Hessler, Lisa Roberts Rapp, Kenneth B. Idler, Chang H. Park, Joel D. Leverson, Lloyd T. Lam

**Affiliations:** AbbVie Oncology, North Waukegan Road North, Chicago, IL 60064-6098 USA

**Keywords:** BCL-2, BCL-X_L_, MCL-1, Apoptosis

## Abstract

**Background:**

Venetoclax (ABT-199), a first-in-class orally bioavailable BCL-2-selective inhibitor, was recently approved by the FDA for use in patients with 17p-deleted chronic lymphocytic leukemia who have received prior therapy. It is also being evaluated in numerous clinical trials for treating patients with various hematologic malignancies. As with any targeted cancer therapy, it is critically important to identify potential mechanisms of resistance, both for patient stratification and developing strategies to overcome resistance, either before it develops or as it emerges.

**Methods:**

In order to gain a more comprehensive insight into the nature of venetoclax resistance mechanisms, we evaluated the changes in the BCL-2 family members at the genetic and expression levels in seven different venetoclax-resistant derived leukemia and lymphoma cell lines.

**Results:**

Gene and protein expression analyses identified a number of different alterations in the expression of pro- and anti-apoptotic BCL-2 family members. In the resistant derived cells, an increase in either or both the anti-apoptotic proteins BCL-X_L_ or MCL-1, which are not targeted by venetoclax was observed, and either concomitant or exclusive with a decrease in one or more pro-apoptotic proteins. In addition, mutational analysis also revealed a mutation in the BH3 binding groove (F104L) that could potentially interfere with venetoclax-binding. Not all changes may be causally related to venetoclax resistance and may only be an epiphenomenon. For resistant cell lines showing elevations in BCL-X_L_ or MCL-1, strong synergistic cell killing was observed when venetoclax was combined with either BCL-X_L_- or MCL-1-selective inhibitors, respectively. This highlights the importance of BCL-X_L_- and MCL-1 as causally contributing to venetoclax resistance.

**Conclusions:**

Overall our study identified numerous changes in multiple resistant lines; the changes were neither mutually exclusive nor universal across the cell lines tested, thus exemplifying the complexity and heterogeneity of potential resistance mechanisms. Identifying and evaluating their contribution has important implications for both patient selection and the rational development of strategies to overcome resistance.

**Electronic supplementary material:**

The online version of this article (doi:10.1186/s12885-017-3383-5) contains supplementary material, which is available to authorized users.

## Background

BCL-2 family proteins function through interactions with each other, and the balance between anti-apoptotic and pro-apoptotic members is critical for preventing or initiating apoptosis [1–3]. This family of proteins share from one to four BCL-2 Homology (BH) motifs. The anti-apoptotic family members, BCL-2, BCL-X_L_, BCL-W, A1 and MCL-1, promote survival by sequestering their pro-apoptotic counterparts such as the “BH3-only” proteins BIM, BAD, BID, HRK, NOXA and PUMA, and the multi-BH3 domain proteins BAK and BAX. BAK and BAX are the ultimate effectors of apoptosis. When free from anti-apoptotic proteins, they can become activated and subsequently oligomerize to form large pores in the mitochondrial outer membrane which enable the release of cytochrome *c* and subsequent activation of the intrinsic apoptosis pathway through a caspase cleavage cascade.

The link between overexpressed anti-apoptotic BCL-2 family proteins and cancer is now well established [4]. Enhanced expression of these proteins has been reported in numerous cancers, which permits cell growth and survival in the presence of apoptotic signals associated with the transformed phenotype, and can also lead to the failure of chemotherapeutic strategies. Navitoclax (ABT-263), an orally bioavailable small-molecule inhibitor of BCL-2, BCL-X_L_, and BCL-W [5], showed signs of clinical antitumor activity in chronic lymphocytic leukemia (CLL). However, most solid tumors are resistant to navitoclax due to high expression of MCL-1, to which the drug has a low affinity [5, 6]. In addition it has been shown that high levels of MCL-1 co-related with resistance to ABT-263 in a panel of leukemia/lymphoma cell lines [6]. Also as predicted by preclinical data, inhibition of BCL-X_L_ by navitoclax induces a rapid, concentration-dependent decrease in the number of platelets [7–9]. This undesirable mechanism-based effect such as thrombocytopenia limited the ability to drive ABT-263 concentrations into a highly efficacious range.

Recently, a unique BCL-2–small molecule cocrystal structure was exploited to guide the rational design of venetoclax (ABT-199), a selective BCL-2 inhibitor intended to circumvent thrombocytopenia associated with BCL-X_L_ inhibition [10]. Venetoclax is a first-in-class orally bioavailable BCL-2-selective inhibitor that has high binding affinity to BCL-2 (K_i_ = <0.01 nM) but not BCL-X_L,_ BCL-W or MCL-1 (K_i_ values = 48 nM, 21 nM and >440 nM, respectively). Venetoclax exhibits single-agent activity against a variety of leukemia/lymphoma cell lines *in vitro* and *in vivo* and clinical activity has been observed in CLL, non-Hodgkin lymphomas (NHL), acute myelogenous leukemia (AML) and multiple myeloma patients treated with venetoclax as a monotherapy [11]. Venetoclax causes substantially less platelet killing *ex vivo* and *in vivo* as compared to navitoclax [10]. In addition to showing preclinical efficacy in BCL-2–dependent cell lines and tumor xenograft models, venetoclax demonstrated immediate antileukemic activity after a single dose in three patients with refractory CLL while causing only minor changes in platelet counts [11]. The results of that phase 1 study and a phase 2 study focused on CLL patients with the high-risk 17p deletion were recently published [11, 12]. Of 116 patients in the phase 1 study, 79% exhibited objective responses to venetoclax, with 20% exhibiting complete responses (CR). Similar overall response rates (ORR) were observed in the 17p-deleted subset of patients in the phase 1 study (71 % ORR) and the phase 2 study dedicated to 17p-deleted CLL (79.4% ORR).

As with any targeted cancer therapy, it is important to identify potential mechanisms of venetoclax resistance, not only to inform patient selection but also to develop strategies to circumvent resistance as it emerges [13]. Previously we demonstrated that MCL-1 overexpression is an inherent resistance factor for ABT-737, a potent BCL-2/BCL-X_L_ inhibitor, in a panel of SCLC cell lines, as well as an acquired resistance factor in H146 cells that had been selected for survival in the presence of ABT-737 [14]. To more fully elucidate the potential mechanisms that may be involved in resistance to venetoclax, we undertook a study to assess potential changes in the expression levels of BCL-2 family members following extended treatment with venetoclax. We generated resistant variants from venetoclax-sensitive cell lines of different leukemia and lymphoma subtypes (two diffuse large B-cell lymphomas, two follicular lymphomas, one leukemia line, and two mantle cell lymphomas) by increasing exposure to venetoclax in a stepwise manner over time. We identified alterations in both pro-apoptotic and anti-apoptotic family members that account for resistance mechanisms. In several cases where the anti-apoptotic BCL-2 relatives MCL-1 or BCL-X_L_ were upregulated, sensitivity to venetoclax could be restored by co-treating with the appropriate MCL-1-selective or BCL-X_L_-selective inhibitor, confirming both are causally related in mediating resistance to venetoclax. These data will help facilitate the rational design of combination strategies to circumvent venetoclax resistance and guide patient selection in future clinical trials.

## Methods

### Reagents

Venetoclax (ABT-199/GDC-0199), navitoclax (ABT-263), BCL-X_L_-selective inhibitor A-1155463 [15], and MCL-1-selective inhibitor A-1208746 [16] were synthesized at AbbVie (North Chicago, IL). All antibodies were purchased from Epitomics (Burlingame, CA).

### Cell culture and cell-based assays

NHL cell lines HBL1, SC-1, U2932, and OCI-Ly1 were cultured in IMDM (Invitrogen Corp., Grand Island, NY) supplemented with 10% human serum (Sigma). All other cell lines were cultured in RPMI supplemented with 10% fetal bovine serum (Invitrogen), 1% sodium pyruvate, and 4.5 g/L glucose (Invitrogen Corp., Grand Island, NY). All cell lines were maintained in a humidified chamber at 37^o^C containing 5% CO_2_. Cells resistant to venetoclax were generated by chronically exposing the parental cells to venetoclax starting at a sub-lethal concentration and subsequently step-wise increasing the concentration over several weeks. Selection pressure was maintained by growing the resistant cells in the presence of at least 1 μM venetoclax.

### Sequencing analysis

For the 14 cell lines, genomic DNA was extracted using the DNeasy blood and tissue kit (Qiagen). DNA was quantified using a Nanodrop spectrophotometer (Thermo Fisher, Waltham, MA). A total of four amplicons covering *BCL2* exons 1 and 2 were sequenced using the primers and cycling conditions (Additional file 1: Table S1). 10 μl of the PCR products were purified using AMPure magnetic beads (Beckman Coulter, Inc., Brea, CA) and eluted in 40 μl of sdH2O. 4 μl of the purified product was cycle sequenced using Big Dye Terminator Mix v3.1(Applied Biosystems, Grand Island, NY) at 1/16 chemistry. Both forward and reverse orientations of the PCR products were sequenced and applied to an Applied Biosystems 3130xl DNA Sequencer (Applied Biosystems, Grand Island, NY). Base calling was performed using Sequence Analysis v5.2 (Applied Biosystems, Grand Island, NY) with the KB basecaller v1.2. Sequence data was aligned to the reference *BCL2* sequence (NM_000633.2) using Sequencher (Gene Codes Corp., Ann Arbor, MI).

### Western blot analysis

Cell lysates were prepared in RIPA buffer (Sigma) plus protease inhibitor cocktail (Roche). 20 μg of total protein was resolved on Bis-Tris gels and transferred to PVDF membranes. Blots were probed overnight at 4^o^C with the appropriate primary antibodies and for 1 hr with the secondary antibodies the following day. Blots were imaged using a LI-COR Biosciences (Lincoln, Nebraska) Odyssey imager.

### Cytotoxicity assay

Cells were treated with indicated agents in 96-well tissue culture plates in RPMI supplemented with 10% FBS for 1 day before assessing viability using the CellTiter Glo Luminescent cell viability assay according to the manufacturer’s protocol (Promega, Madison, WI). Synergistic activities of venetoclax and specific MCL-1 or BCL-X_L_ inhibitors were determined using the Bliss additivity model, whereby the combined response (C) of both agents with individual effects A and B is C = A + B – (A × B), where A and B represent the fractional inhibition between 0 and 1. Drug interactions were considered synergistic if the combined Bliss scores were >0 and antagonistic if <0 [17]. Student’s t-test was used to determine statistical significance.

### Microarrays

RNA was converted to biotinylated antisense RNA and hybridized to Affymetrix human U133A 2.0 gene expression arrays. Data were analyzed using Rosetta Resolver software comparing each venetoclax-resistant cell line to its untreated parental line. Genes/probe sets with >1.5-fold change relative to parental and meeting a 5% False Discovery Rate were considered significant. Zero signifies no significant change from baseline.

### DNA copy number determination

DNA was amplified, biotin labeled, and then hybridized to Affymetrix SNP 6.0 arrays. Gene copy numbers were calculated after segment smoothing of raw data using Partek software.

### Flow cytometry

Approximately 1-2×10^6^ cells were pelleted by centrifugation, resuspended in Lyse Fix Buffer I (BD BioSciences) for 10 minutes, washed once with PBS-D, then permeabilized with 1x BD Phosflow Perm Wash Buffer I. BAX antibody or isotype control was added to 1×10^5^ fixed/permeabilized cells. Samples were incubated for 30 min at room temperature in the dark and washed, followed by the addition of an APC-conjugated F(ab’)2. After incubation for 30 min at room temperature, the cells were washed twice and the samples stored in Fix buffer I. Samples were then analyzed using a BD FACSCalibur and CellQuest Pro software [18].

## Results

### Generating resistance to venetoclax in B-cell leukemia and lymphoma cells

**Fig. 1:**
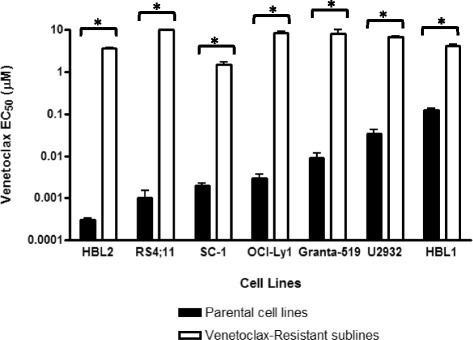
Generation of venetoclax-resistant cancer cell lines. Hematologic cancer cells lines were incubated in gradually increasing concentrations of venetoclax to isolate populations resistant to μM concentrations. Cell killing EC_50_ values are plotted for parental cell lines and the venetoclax-resistant sublines. Data are presented as the mean +/- S.E.M. of three independent experiments. Asterisks denote p < 0.05

We generated cells resistant to venetoclax (199R) in six lymphoma lines, including two ABC subtype DLBCL cell lines (HBL1 and U2932), two follicular/GCB lymphoma cell lines (OCI-Ly1 and SC-1), two mantle cell lymphoma cell lines (HBL2 and Granta-519) as well as one leukemia line (RS4;11). These cell lines were chosen because they are highly sensitive to venetoclax-mediated killing, with EC_50_ values ˂1 μM. These cell lines were exposed to low (nM) doses of venetoclax for short periods of time (up to three months). Once sufficient numbers of stably viable cells were obtained, the dose was increased. After several months of treatment, cells were capable of maintaining viability with continuous exposure to venetoclax at >1μM for SC-1 199R and >3μM for HBL1 199R, U2932 199R, OCI-Ly1 199R, HBL2 199R, Granta-519 199R, and RS4;11 199R cells (Fig. 1).

### Changes in gene and protein expression in venetoclax-resistant lymphoma and leukemia cell lines

We first investigated whether venetoclax resistance was accompanied by changes in the gene expression pattern of *BCL2* family members using microarray analysis. Gene expression analysis indicated that no single change occurred in all cases. Instead, a variety of *BCL2* family genes showed expression changes across this panel of cell lines. For example, there was an increase of *BCL2L1* (BCL-X_L_) expression in SC-1 199R cells, a decrease of *BAX* in RS4;11 199R and HBL1 199R cells, a decrease of *BIM* in HBL1 199R, OCI-Ly1 199R, and SC-1 199R cells, an increase of *MCL1* in OCI-Ly1 199R, an increase of *BCL2A1* in HBL-2 199R cells, and a decrease of *PMAIP1* (NOXA) in multiple cell lines (Table 1). In some cases, multiple alterations were observed within the same cell population, potentially due to the presence of multiple clones.

**Table 1:** Change in *BCL2* family member mRNA levels in venetoclax-resistant cells relative to parental cells. Gene expression data were analyzed using Rosetta Resolver software comparing each venetoclax-resistant cell line to its untreated parental line. Genes/probe sets with > 1.5-fold change relative to parental and meeting a 5% False Discovery Rate were considered significant. Zero signifies no significant change from baseline

		Cell lines						
Primary Sequence Name	Protein Name	OCI-Ly1 199R	SC-1 199R	HBL1 199R	U2932 199R	RS4;11 199R	HBL2 199R	Granta-519 199R
*BCL2*	BCL-2	-2.20	1.25	0.00	-3.35	0.00	-13.11	0.00
*BCL2L1*	BCL-X_L_	0.00	14.88	0.00	-2.24	0.00	0.00	0.00
*MCL1*	MCL-1	2.20	1.91	0.00	-1.89	0.00	-1.71	0.00
*BCL2A1*	BCL-A1	-6.13	0.00	0.00	0.00	0.00	2.19	0.00
*BCL2L2*	BCL-W	0.00	0.00	0.00	0.00	0.00	0.00	0.00
*BAX*	BAX	-1.83	0.00	-2.43	0.00	-3.50	0.00	0.00
*BAK1*	BAK	0.00	0.00	0.00	0.00	0.00	0.00	0.00
*BID*	BID	0.00	0.00	0.00	0.00	0.00	0.00	0.00
*BCL2L11*	BIM	-3.33	-3.22	-16.70	0.00	0.00	0.00	0.00
*PMAIP1*	NOXA	-2.54	-1.50	-2.19	-1.65	0.00	-3.16	0.00
*BBC3*	PUMA	-1.64	-2.08	0.00	3.49	0.00	0.00	0.00
*BAD*	BAD	1.34	0.00	2.40	0.00	0.00	0.00	0.00

**Fig. 2.**
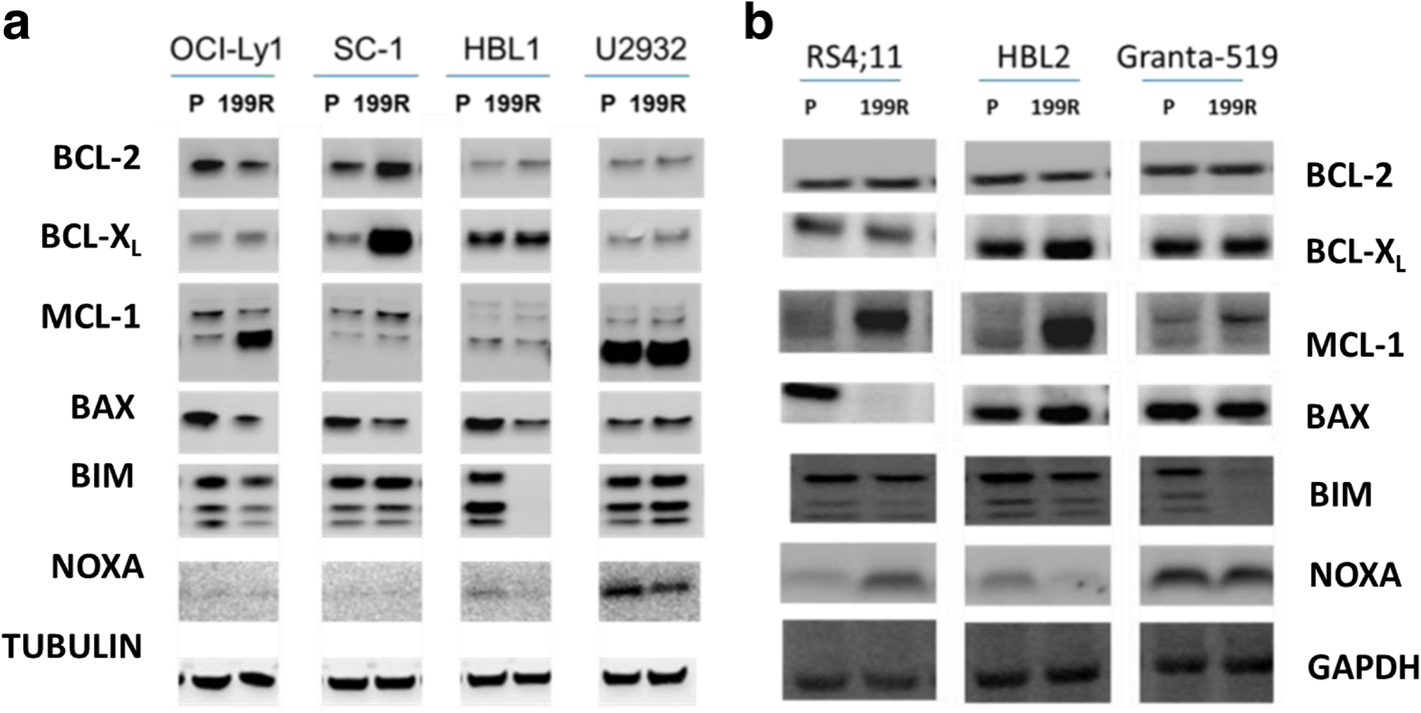
BCL-2 family protein expression in parental cell lines and venetoclax-resistant populations. Equivalent amounts of protein from whole cell lysates generated from parental cell lines and their venetoclax-resistant sublines growing in (**a**) human serum and (**b**) fetal bovine serum were assessed by immunoblotting for BCL-2 family proteins as indicated

To determine whether these gene expression changes translated into altered protein levels, we next performed immunoblotting analysis of BCL-2 family members in these cell lines (Fig. 2a, b). In general, we found that protein expression changes recapitulated the mRNA results. For example, we observed increased MCL-1 in OCI-Ly1 199R cells, decreased BAX in RS4;11 199R and HBL1 199R cells, decreased NOXA in HBL1 199R and HBL2 199R cells, decreased BIM in HBL1 199R and OCI-Ly1 199R cells, and increased BCL-X_L_ in SC-1 199R cells. We also observed changes in protein expression without changes in mRNA expression. For example, MCL-1 protein levels were elevated in HBL2 199R and Granta-519 199R cells without a corresponding elevation in *MCL1* transcript. Similar results were observed using a quantitative Luminex assay to measure the modulation of the expression of anti-apoptotic proteins BCL-2, BCL-X_L_, and MCL-1 [19]. Interestingly, from the western blots there did appear to be an increase of MCL-1 expression in U2932 199R cells. U2932 cells normally express high basal levels of MCL-1 mRNA and protein, and so the signal may have been saturated in the immunoblotting analysis. Using the Luminex assay we confirmed that there was a 2-fold increase in MCL-1 levels in the resistant U2932 cells (Additiona file 1: Figure S1).

To investigate changes at the DNA level, we evaluated the copy number of these genes (Table 2). Our analysis identified a loss of *BIM* in HBL1 199R cells, a gain in *MCL1* in OCI-Ly1 199R and SC-1 199R cells, a gain in *BCL2L1* in SC-1 199R cells, and loss of *BCL2* and *PMAIP1* in U2932 199R and HBL2 199R cells. The simultaneous loss of *BCL2* and *PMAIP1* may be due to the proximity of these two loci on chromosome 18 [20]. No obvious changes were observed in RS4;11 199R or Granta-519 199R cells (Table 2). Taken together, there is no one particular mechanism common to all the resistant variants but instead multiple mechanisms that could potentially lead to venetoclax resistance. Some changes involved alterations at the gene level while others involved post-translational regulation.

**Table 2:** DNA copy number of *BCL2* family genes in parental cell lines and venetoclax-resistant populations. Key changes are highlighted in bold

	Cell Lines													
Primary Sequence Name	OCI-Ly1 parent	OCI-Ly1 199R	SC-1 parent	SC-1 199R	HBL1 parent	HBL1 199R	U2932 Parent	U2932 199R	RS4;11 parent	RS4;11 199R	HBL2 parent	HBL2 199R	Granta-519 parent	Granta-519 199R
***BCL2***	0.46	0.46	6.00	**7.79**	3.01	3.19	14.86	**7.08**	2.05	1.97	6.07	**1.34**	5.99	5.61
***BCL2L1***	2.08	2.27	2.17	**9.16**	2.08	2.03	2.09	2.14	2.06	2.05	1.93	1.99	1.83	1.85
***MCL1***	2.07	**3.21**	2.18	**5.12**	2.11	2.04	9.99	**11.25**	2.16	2.10	2.99	1.96	2.39	2.40
***BCL2A1***	2.04	2.08	2.10	2.07	2.08	2.03	3.05	2.88	2.00	2.03	1.91	1.91	2.33	2.05
***BCL2L2***	2.06	2.15	2.11	2.10	2.08	2.05	2.08	2.10	2.18	2.14	1.95	1.99	1.30	1.29
***BAX***	2.09	2.22	2.18	2.00	2.17	2.04	3.12	2.92	2.17	2.18	2.06	1.99	2.48	2.43
***BAK1***	2.06	2.17	2.23	2.12	1.13	1.01	2.16	2.16	2.12	2.11	2.02	2.10	1.94	1.93
***BID***	2.07	2.23	2.30	2.18	2.21	2.08	2.18	2.05	2.24	2.20	2.04	2.11	1.98	2.13
***BCL2L11***	2.02	2.10	2.46	2.02	2.10	**0.46**	1.13	2.01	1.99	1.99	1.97	2.21	1.93	2.12
***PMAIP1***	1.99	1.55	3.07	2.26	3.12	3.15	18.25	**9.50**	2.05	1.97	3.76	**1.26**	6.22	**5.61**
***BBC3***	2.09	2.22	2.24	2.00	2.17	2.04	3.12	2.92	2.17	2.18	2.06	1.99	2.41	2.43
***BAD***	2.01	2.11	2.36	2.18	2.07	2.05	2.11	2.07	2.12	2.12	2.05	2.00	1.86	1.60

### Selection of pre-existing sub-clones of RS4;11 cells with low or no BAX expression leads to resistance to venetoclax

**Fig. 3.**
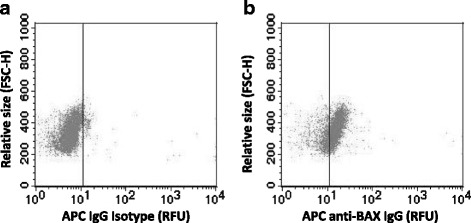
Sub-population of parental RS4;11 cells lack BAX expression. BAX protein levels were quantified in parental RS4;11 cells by quantitative flow cytometry. **a** Dot plot of naïve parental RS4;11 cells stained with APC-isotype control. **b** Dot plot of naïve parental RS4;11 cells stained for BAX. Data are representative of three separate experiments

While multiple lymphoma cell lines showed modulation in the copy number of *BCL2* family genes, the means by which RS4;11 199R cells lost BAX expression without a loss of the *BAX* gene locus is unknown. We hypothesized that subclones of RS4;11 cells with low or no BAX expression may exist in the original venetoclax-sensitive population. To test this hypothesis, RS4;11 cells were stained with APC-isotype control and BAX antibodies and evaluated on a cell-by-cell basis for BAX expression using flow cytometry (Fig. 3a, b). Surprisingly, 21% of the naïve parental RS4;11 cells had no detectable BAX or very low BAX expression (Fig. 3b). Thus, low exposure with venetoclax may have favored the selection of a BAX-deficient population.

### Mutation in the BH3-binding domain of BCL2 confers resistance to venetoclax

**Fig. 4.**
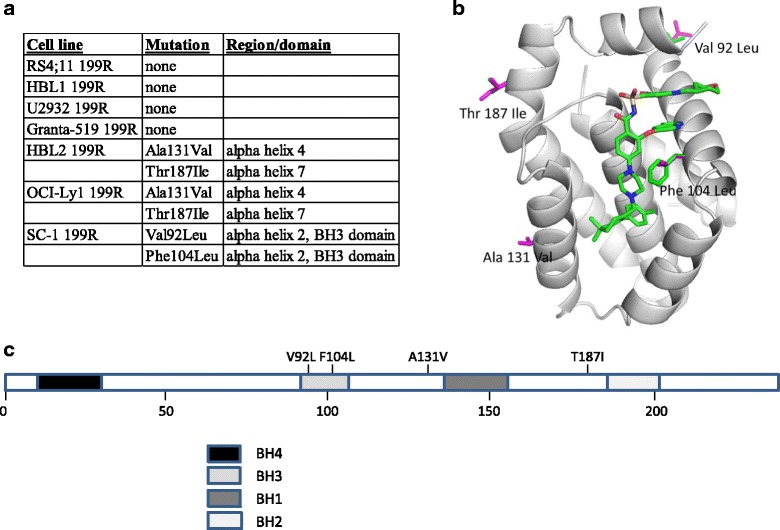
Mutation in the BH3-binding domain of BCL-2 in SC-1 cells confer resistance to venetoclax. **a** Table showing region/domain mutations reside in the cell lines identified by sequencing. **b** X-ray crystal structure of BCL-2 with F104L mutation, which is predicted to lower affinity for venetoclax. **c** General domain structure of BCL2 showing the amino acids mutationally altered in DLBCL. A, Ala; F, Phe; I, Ile; L, Lys; T, Thr; V, Val

Venetoclax competitively inhibits the interaction between BCL-2 and pro-apoptotic proteins through binding to the BH3-binding groove of BCL-2. Thus, one potential mechanism of resistance is through the acquisition of BH3-binding groove mutations that decrease affinity to venetoclax. Such mutations (F101C and F101L) were recently identified in a mouse B-cell lymphoma cell line that had been selected for venetoclax resistance [21]. Upon sequencing *BCL2* in each pair of parental and resistant cell lines, we found that four of the resistant lines retained the parental sequence and three had nonsynonymous mutations (Fig. 4a). SC-1 199R cells possessed the same F104L mutation in the BH3 domain that had been identified previously to affect venetoclax binding [21]. Another mutation V92L was also identified to reside in the BH3 domain of BCL2 in SC-1 199R cells. However, since V92L is outside the binding groove for venetoclax, it should not affect venetoclax binding (Fig. 4b). The other two mutations (A131V and T187I) are outside any functionally essential domains (such as the BH domains) and are likely passenger mutations (Fig. 4c) [22].

### MCL-1 and BCL-X_L_ inhibition increases sensitivity in resistant cells

**Fig. 5.**
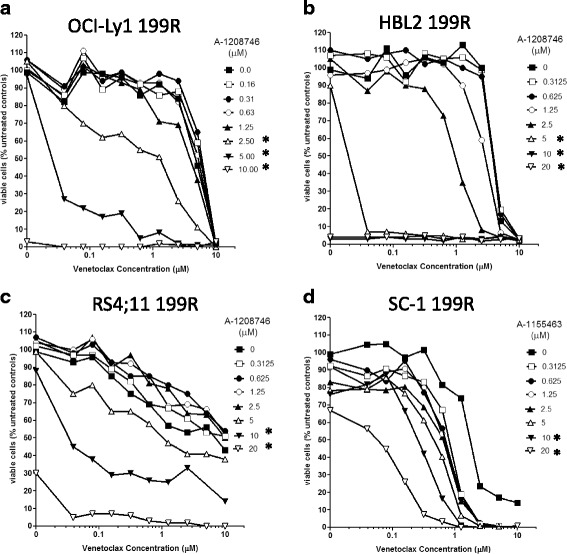
Combination of MCL-1- or BCL-X_L_-selective inhibitors resensitizes venetoclax-resistant cells to venetoclax. Venetoclax-resistant (199R) cells were incubated in varying concentrations of venetoclax with or without increasing concentrations of the MCL-1-selective inhibitor A-1208746 (**a-c**) or the BCL-X_L_-selective inhibitor A**-**1155463 (d) for 24 hours before assessing cell viability. Data are presented as the percent viability compared to untreated cells. Bliss scores were calculated as described in Materials and Methods. Data are representative of three separate experiments. Asterisk designates significant difference (p<0.05) as compared to venetoclax-treatment alone.

Relative to their parental counterparts, several resistant variants showed increased expression of the BCL-2 relatives MCL-1 or BCL-X_L_. RS4;11 199R, HBL2 199R, and OCI-Ly1 199R cells exhibited a significant increase of MCL-1 expression, whereas elevated BCL-X_L_ expression was observed in SC-1 199R cells. To test the functional relevance of these changes, we next combined venetoclax with the MCL-1-selective inhibitor A-1208746 or BCL-X_L_-selective inhibitor A-1155463 (Fig. 5). Strong synergy (Bliss sum equal to 193, 809 and 804) was observed in RS4;11 199R, HBL2 199R, and OCI-Ly1 199R cells, respectively, following cotreatment with venetoclax (<0.1 μM) and the MCL-1 inhibitor A-1208746 (between 2.5-5.0 μM) (Fig. 5a-c). Similarly, strong synergy (Bliss sum equal to 1121) was observed in the SC-1 199R cells cotreated with venetoclax and BCL-X_L_ inhibitor A-1155463 (Fig. 5d). These data confirm that MCL-1 or BCL-X_L_ can play a compensatory role in mediating resistance to venetoclax and point to potential combination strategies for overcoming this mode of resistance.

## Discussion

In this study, we generated leukemia and lymphoma cell line populations resistant to venetoclax by selection in the presence of gradually increasing drug concentrations. These cells were then compared to their parental counterparts for obvious changes in *BCL2* family gene sequences or copy number, *BCL2* family transcript levels, and/or the expression of BCL-2 family proteins to gain a better understanding of the mechanisms of resistance to venetoclax.

Notably, we found that a variety of distinct mechanisms had the potential to confer venetoclax resistance. While no single change accounted for resistance in all the cell pairs, some common themes emerged, such as increases in redundantly acting anti-apoptotic proteins like MCL-1 (OCI-Ly1, RS4;11, Granta-519, HBL2 and U2932) or BCL-X_L_ (SC-1). In these populations, resistance to venetoclax could be reversed by concurrent treatment with the MCL-1-selective inhibitor A-1208746 or the BCL-X_L_-selective inhibitor A-1155463, demonstrating that these proteins are directly involved in mediating the resistance and highlighting the importance of developing BCL-X_L_ and MCL-1 inhibitors for clinical use. It should be noted that A-1208746 typically shows cell killing around 2.5-10 μM for MCL-1-dependent cell lines [23]. Therefore, the concentrations at which combination effects are observed are consistent with the on-target activity of A-1208746. Once MCL-1 is sufficiently neutralized, the EC_50_ for venetoclax shifted to below 0.1 μM (Fig. 5). Changes in pro-apoptotic proteins were also observed in multiple resistant cell lines. In these cases, decreases were observed. Multiple cell lines showed reductions in the BH3-only protein BIM (Granta-519, HBL1, OCI-Ly1) or the effector protein BAX relative to their parental counterpart. A striking absence of BAX was observed in venetoclax-resistant RS4;11 cells and subsequent flow cytometry analyses identified a pre-existing sub-population of BAX-deficient cells amongst the parental RS4;11 population, thus representing a possible case of innate resistance to venetoclax.

Changes in the expression of apoptotic proteins were sometimes associated with gene copy number gains or losses and/or changes in mRNA levels. In the most striking examples, venetoclax-resistant SC-1 cells showed amplification of the gene locus encoding BCL-X_L_ (*BCL2L1*) and a corresponding elevation in *BCL2L1* mRNA and BCL-X_L_ protein, whereas venetoclax-resistant HBL1 cells showed a loss in the gene encoding BIM (*BCL2L11*) with corresponding reduction in *BCL2L11* transcript and absence of detectable BIM protein. Recently, it was shown that PI3Kδ inhibitor-induced apoptosis is BIM-mediated as Bim^-/-^ Eμ-Tcl1 Tg leukemias demonstrated resistance [24]. In some cases, *BCL2* family transcripts were elevated without evidence of a corresponding gene gain or amplification, suggesting that altered signaling pathways may account for elevated protein levels. Elevated MCL-1 levels were sometimes associated with elevated *MCL1* mRNA (OCI-Ly1), and sometimes not (U2932, HBL2 and Granta-519), indicating that alterations in pathways impacting MCL-1 stability may also be involved. Although the originating source of the resistance signal was not immediately apparent in these cases, the most target-proximal cause of resistance is likely to be the altered expression of the BCL-2 family protein furthest downstream in the intrinsic cell death pathway.

Most proximal of all, mutations in the BH3-binding groove of BCL-2 itself were also identified in venetoclax-resistant cell populations, including the F104L mutation, which was previously shown to reduce venetoclax binding affinity and confer resistance [21]. Such mutations have yet to be reported in association with clinical resistance to venetoclax. Nevertheless, should this mechanism arise as a significant factor in the development of venetoclax resistance, it should be possible to synthesize BCL-2 inhibitors that maintain high affinity for F104L-mutated BCL-2, akin to the second- and third-generation tyrosine kinase inhibitors that were designed in response to the Abl kinase gatekeeper mutations found to confer imatinib resistance[25].

It should also be noted that the concentrations up to which these cells show resistance to venetoclax are clinically relevant. Venetoclax can achieve and maintain plasma exposures ~1-3 μM at daily doses ranging from 400 mg to 800 mg [12]. Finally, although numerous ABT-199-resistant cell line populations were isolated in this study, we cannot make distinctions between cells that acquired resistance by “rewiring” and cases where pre-existing sub-clones with constitutive resistance were selected for. The identification of a pre-existing sub-population of BAX-deficient cells amongst the parental RS4;11 population may represent an example of the latter; however, this could not be formally demonstrated as cell fixation was required to identify the population lacking BAX.

## Conclusions

The data presented here indicate that diverse mechanisms have the potential to contribute to venetoclax resistance. However, most have in common a clear alteration in the expression of at least one pro-apoptotic or anti-apoptotic BCL-2 family protein that is likely to have a strong impact on the action of venetoclax. Although mechanisms of venetoclax resistance have yet to be defined in the clinic, the findings presented here suggest a number of hypotheses that can be explored. Hopefully these studies will provide a basis for further investigation and will enable the research community to develop the next generation of therapeutics that can reverse or circumvent resistance to this promising therapeutic.

## Additional file

Additional file 1: Table S1. PCR primers and conditions. **Figure S1**. MCL-1 protein expression in parental cell lines and venetoclax-resistant populations. MCL-1 protein expression was measured using an assay developed based on the Luminex technology [19]. In brief, MCL-1 capture antibody (Santa Cruz Biotechnology Inc., Santa Cruz, CA) was custom-conjugated to Luminex carboxyl beads (bead region 9) by Millipore (St. Charles, MO, USA). MCL-1 detection antibody (Santa Cruz Biotechnology Inc.) was also conjugated to biotin through a custom service provided by Millipore. Cells were lysed in MILLIPLEX MAP lysis buffer 1 (Millipore Cat. no. 43-040, Danvers, MA, USA) containing protease inhibitor cocktail (Sigma). Data are presented as median fluorescent intensity (MFI). Equivalent amounts of protein from whole cell lysates generated from parental cell lines and their venetoclax-resistant versions were assessed. The signal was read using a Luminex FlexMap 3D system (Luminex, Austin, TX). Asterisks denote p < 0.05. (PPTX 79 kb)

## References

[1] Adams JM, Cory S (2007). The Bcl-2 apoptotic switch in cancer development and therapy. Oncogene.

[2] Czabotar PE, Lessene G, Strasser A, Adams JM (2014). Control of apoptosis by the BCL-2 protein family: implications for physiology and therapy. Nat Rev Mol Cell Biol.

[3] Danial NN, Korsmeyer SJ (2004). Cell death: critical control points. Cell.

[4] Lam LT, Zhang H, Chyla B (2012). Biomarkers of Therapeutic Response to BCL2 Antagonists in Cancer. Mol Diagn Ther.

[5] Tse C, Shoemaker AR, Adickes J, Anderson MG, Chen J, Jin S, et al. ABT-263: a potent and orally bioavailable Bcl-2 family inhibitor. *Cancer Res*. 2008;68:3421–8. 10.1158/0008-5472.CAN-07-5836 18451170

[6] Tahir SK, Wass J, Joseph MK, Devanarayan V, Hessler P, Zhang H, et al. Identification of expression signatures predictive of sensitivity to the Bcl-2 family member inhibitor ABT-263 in small cell lung carcinoma and leukemia/lymphoma cell lines. *Mol Cancer Ther*. 2010;9:545–57. 10.1158/1535-7163.MCT-09-0651 20179162

[7] Gandhi L, Camidge DR, Ribeiro de Oliveira M, Bonomi P, Gandara D, Khaira D, et al. Phase I study of Navitoclax (ABT-263), a novel Bcl-2 family inhibitor, in patients with small-cell lung cancer and other solid tumors. *J Clin Oncol*. 2011;29:909–16. 10.1200/JCO.2010.31.6208 PMC4668282 21282543

[8] Wilson WH, O'Connor OA, Czuczman MS, LaCasce AS, Gerecitano JF, Leonard JP, et al. Navitoclax, a targeted high-affinity inhibitor of BCL-2, in lymphoid malignancies: a phase 1 dose-escalation study of safety, pharmacokinetics, pharmacodynamics, and antitumour activity. *Lancet Oncol*. 2010;11:1149–59. 10.1016/S1470-2045(10)70261-8 PMC3025495 21094089

[9] Rudin CM, Hann CL, Garon EB, Ribeiro de Oliveira M, Bonomi PD, Camidge DR, et al. Phase II study of single-agent navitoclax (ABT-263) and biomarker correlates in patients with relapsed small cell lung cancer. *Clin Cancer Res*. 2012;18:3163–9. 10.1158/1078-0432.CCR-11-3090 PMC3715059 22496272

[10] Souers AJ, Leverson JD, Boghaert ER, Ackler SL, Catron ND, Chen J, et al. ABT-199, a potent and selective BCL-2 inhibitor, achieves antitumor activity while sparing platelets. *Nat Med*. 2013;19:202–8. 10.1038/nm.3048 23291630

[11] Ng SY, Davids MS (2014). Selective Bcl-2 inhibition to treat chronic lymphocytic leukemia and non-Hodgkin lymphoma. Clin Adv Hematol Oncol.

[12] Roberts AW, Davids MS, Pagel JM, Kahl BS, Puvvada SD, Gerecitano JF, et al. Targeting BCL2 with Venetoclax in Relapsed Chronic Lymphocytic Leukemia. *N Engl J Med*. 2016;374:311–22. 10.1056/NEJMoa1513257 PMC7107002 26639348

[13] Garraway LA, Janne PA (2012). Circumventing cancer drug resistance in the era of personalized medicine. Cancer Discov.

[14] Tahir SK, Yang X, Anderson MG, Morgan-Lappe SE, Sarthy AV, Chen J, et al. Influence of Bcl-2 family members on the cellular response of small-cell lung cancer cell lines to ABT-737. *Cancer Res*. 2007;67:1176–83. 10.1158/0008-5472.CAN-06-2203 17283153

[15] Tao ZF, Hasvold L, Wang L, Wang X, Petros AM, Park CH, et al. Discovery of a Potent and Selective BCL-XL Inhibitor with in Vivo Activity. *ACS Med Chem Lett*. 2014;5:1088–93. 10.1021/ml5001867 PMC4190639 25313317

[16] Bruncko M, Wang L, Sheppard GS, Phillips DC, Tahir SK, Xue J, et al. Structure-guided design of a series of MCL-1 inhibitors with high affinity and selectivity. *J Med Chem*. 2015;58:2180–94. 10.1021/jm501258m 25679114

[17] Borisy AA, Elliott PJ, Hurst NW, Lee MS, Lehar J, Price ER, et al. Systematic discovery of multicomponent therapeutics. *Proc Natl Acad Sci U S A*. 2003;100:7977–82. 10.1073/pnas.1337088100 PMC164698 12799470

[18] Smith ML, Chyla B, McKeegan E, Tahir SK. Development of a Flow Cytometric Method for Quantification of BCL-2 Family Members in Chronic Lymphocytic Leukemia and Correlation with Sensitivity to BCL-2 Family Inhibitors. *Cytometry B Clin Cytom*. 2016; 10.1002/cyto.b.21383 27177607

[19] Lam LT, Roberts-Rapp L (2014). Multiplex analysis of anti-apoptotic BCL2 family and caspase 3 activation by microbead arrays. Assay Drug Dev Technol.

[20] Olejniczak ET, Van Sant C, Anderson MG, Wang G, Tahir SK, Sauter G, et al. Integrative genomic analysis of small-cell lung carcinoma reveals correlates of sensitivity to bcl-2 antagonists and uncovers novel chromosomal gains. *Mol Cancer Res*. 2007;5:331–9. 10.1158/1541-7786.MCR-06-0367 17426248

[21] Fresquet V, Rieger M, Carolis C, Garcia-Barchino MJ, Martinez-Climent JA (2014). Acquired mutations in BCL2 family proteins conferring resistance to the BH3 mimetic ABT-199 in lymphoma. Blood.

[22] Petros AM, Olejniczak ET, Fesik SW (2004). Structural biology of the Bcl-2 family of proteins. Biochim Biophys Acta.

[23] Leverson JD, Zhang H, Chen J, Tahir SK, Phillips DC, Xue J, et al. Potent and selective small-molecule MCL-1 inhibitors demonstrate on-target cancer cell killing activity as single agents and in combination with ABT-263 (navitoclax). *Cell Death Dis*. 2015;6:e1590. 10.1038/cddis.2014.561 PMC4669759 25590800

[24] Carter MJ, Cox KL, Blakemore SJ, Turaj AH, Oldham RJ, Dahal LN, Tannheimer S, Forconi F, Packham G, Cragg MS: PI3Kdelta inhibition elicits anti-leukemic effects through Bim-dependent apoptosis. Leukemia. 2016. https://www.ncbi.nlm.nih.gov/pubmed/27843137. 10.1038/leu.2016.333 PMC5467045 27843137

[25] Jabbour E, Kantarjian H, Cortes J (2015). Use of second- and third-generation tyrosine kinase inhibitors in the treatment of chronic myeloid leukemia: an evolving treatment paradigm. Clin Lymphoma Myeloma Leuk.

