# Supplementary figures and images for: Potential mechanisms of resistance to venetoclax and strategies to circumvent it
Source: BMC Cancer. 2017 Jun 2;17:399. doi: 10.1186/s12885-017-3383-5 (PMC5457565; doi:10.1186/s12885-017-3383-5)

## Slide 1
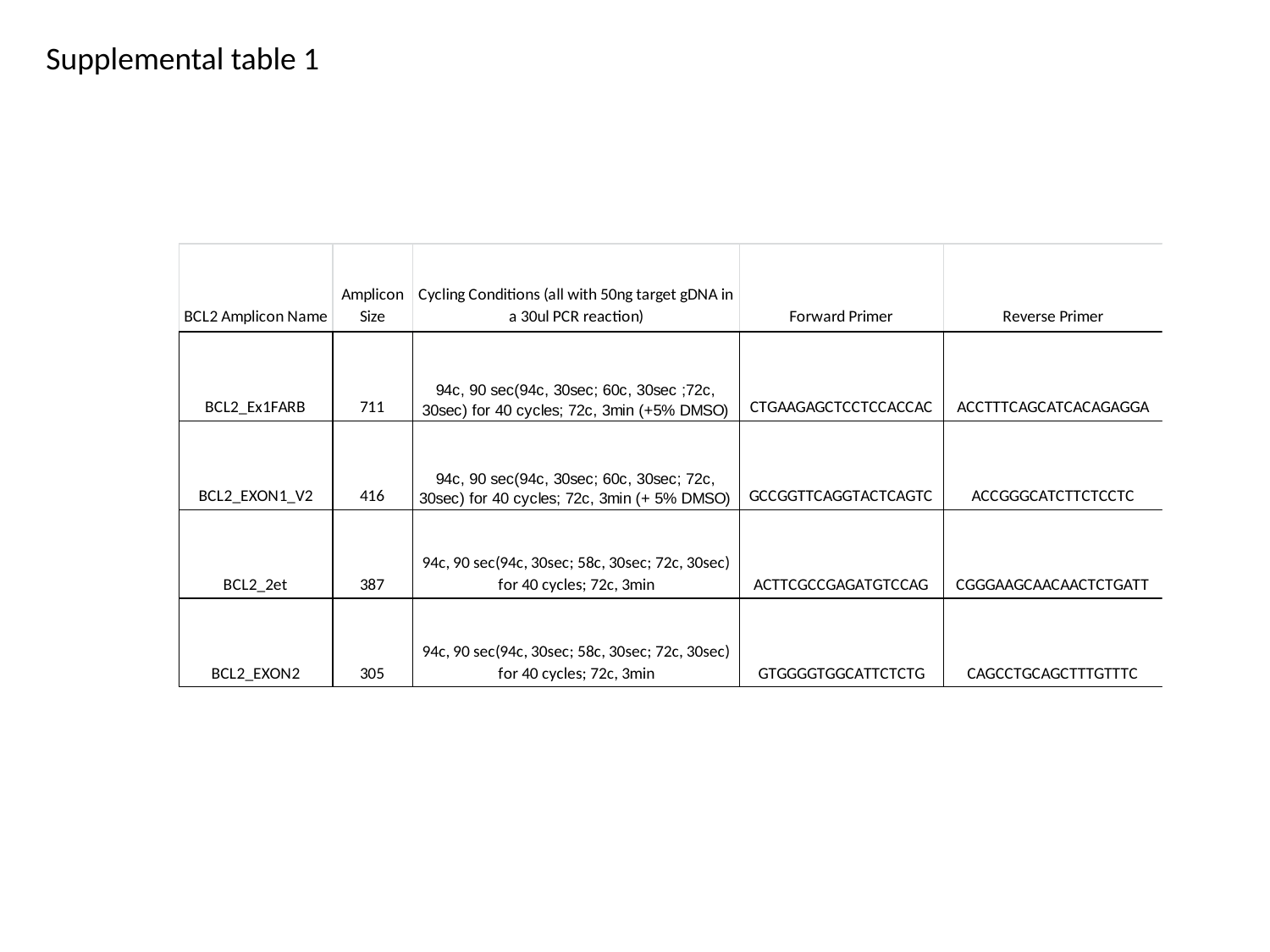

Supplemental table 1

## Slide 2
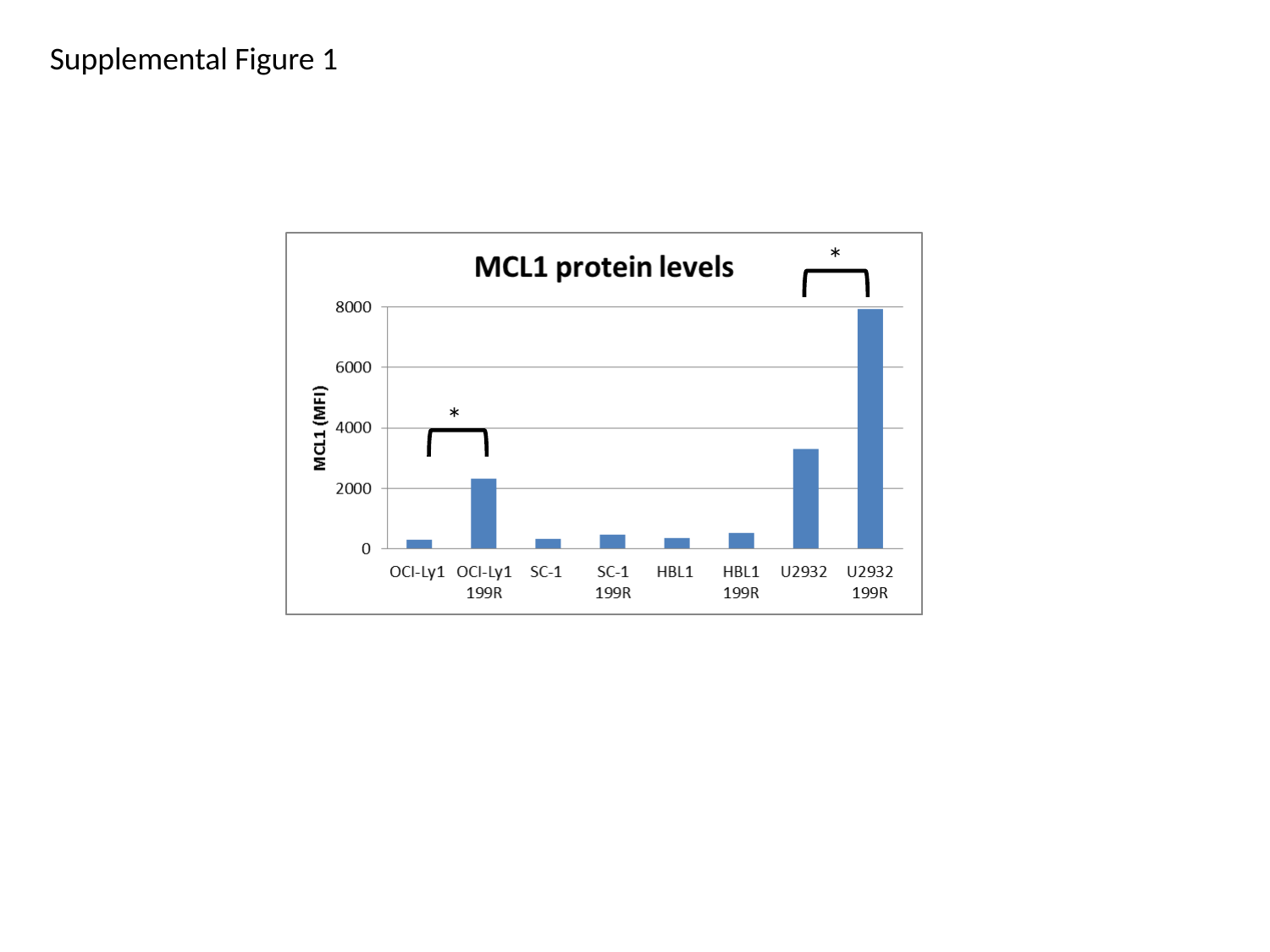

Supplemental Figure 1
*
*

Supplement: Additional file 1: Table S1. — PCR primers and conditions. Figure S1. MCL-1 protein expression in parental cell lines and venetoclax-resistant populations. MCL-1 protein expression was measured using an assay developed based on the Luminex technology [19]. In brief, MCL-1 capture antibody (Santa Cruz Biotechnology Inc., Santa Cruz, CA) was custom-conjugated to Luminex carboxyl beads (bead region 9) by Millipore (St. Charles, MO, USA). MCL-1 detection antibody (Santa Cruz Biotechnology Inc.) was also conjugated to biotin through a custom service provided by Millipore. Cells were lysed in MILLIPLEX MAP lysis buffer 1 (Millipore Cat. no. 43-040, Danvers, MA, USA) containing protease inhibitor cocktail (Sigma). Data are presented as median fluorescent intensity (MFI). Equivalent amounts of protein from whole cell lysates generated from parental cell lines and their venetoclax-resistant versions were assessed. The signal was read using a Luminex FlexMap 3D system (Luminex, Austin, TX). Asterisks denote p < 0.05. (PPTX 79 kb) [file 12885_2017_3383_MOESM1_ESM.pptx]
